# Continuous vs intermittent Non-Invasive blood pressure MONitoring in preventing postoperative organ failure (niMON): study protocol for an open-label, multicenter randomized trial

**DOI:** 10.1186/s44158-024-00142-w

**Published:** 2024-02-06

**Authors:** Alberto Noto, Athanasios Chalkias, Fabiana Madotto, Lorenzo Ball, Elena Giovanna Bignami, Maurizio Cecconi, Fabio Guarracino, Antonio Messina, Andrea Morelli, Pietro Princi, Filippo Sanfilippo, Sabino Scolletta, Luigi Tritapepe, Andrea Cortegiani, SIAARTI Study Group, Adrian Mira, Agrippino Bellissima, Andrea Puppo, Alberto Galvano, Alessandro Bristot, Alice Scannella, Alicia Ruiz-Escobar, Alicia Sanchez Duran, Andrea Micalef, Andrea Paolo Tramonte, Andreaserena Recchia, Angela Zumpano, Angelica Ancona, Anna Cicalò, Anna Monardo, Annateresa Mazzeo, Antonio David, Antonio Gallina, Antonio Magri, Antonio Pili, Barbara Bifarini, Barbara Franzoso, Beatrice Milan, Begona Ortega Eguiluz, Catalina Puigserver Martorell, Cecilia Novazzi, Celeste Cataldo, Chiara Fiandra, Chiara Merenda, Chiara Nicocia, Clara Bordes García, Claudia Crimi, Claudia Frigieri, Consuelo Brogno, Consuelo Coppola, Cristina Inoriza Nadal, Dario Rocca, David Beniguria, David Pestaña, Davide Ottolina, Guido Di Gregorio, Diego Dominguez Flores, Diego Fiume, Domenico Russo, Eduardo Martin, Elena Alberti, Elena Conesa Lopez, Eleni Laou, Eliana Ximena Montsalve Ortiz, Emma Franceschi, Enrico Polati, Ernesto Trimarchi, Eros Gambaretti, Fabio Gori, Fabrizia Gentili, Federica Albanese, Federica Pennavaria, Filippo Benedetto, Flavia Falchetti, Florinda Messina, Gabriele Finco, Gabriele Selmo, Gabriele Recanelli, Gaetano Gallese, Giacomo Brondi, Giaime Putzu, Giovanna Semeraro, Giovanna Braga, Giulia Pedini, Giulia Torregiani, Giulio Genoese, Giuseppe Calabrese, Giuseppe Stagliano, Giuseppe Servillo, Gloria Zaffaroni, Grazia De Angelis, Graziano Leonardi, Guillermo Ferrer, Ines Verge Montano, Inmaculada Chinchurreta Barquero, Ivana Brunetti, Ivana Pezzoli, Jacopo Rama, Javier Ripollés Melchor, Javier Silva Garcia, Jessica Giuseppina Maugeri, Jesus Garcia Arranz, Juan Vidaldiaz, Katia Audisio, Katia Donadello, Laura Camici, Laura Frigo, Leonardo Gottin, Luana Mollura, Luca Guzzetti, Lucia dei Rio Prieto, Lucia Di Falco, Lucia Tisner Yague, Luciana Raciti, Luigi Cardia, Luis Santè Serna, Manuela Nicastro, Marco Anderloni, Marco Covotta, Maria Loreto, Maria de la Cruz Navas, Maria de los Angeles Chercoles Ruiz, Maria Guisasola Rabés, Mariachiara Ippolito, Marta Giordano, Martina Campione, Massimiliano Giardina, Massimiliano Paltenghi, Massimo Pisanti, Matteo Procopio, Maura Mancini, Melissa Carollo, Michela Massaro, Michele Chianchella, Nicola Logrieco, Nicolas Adam, Nicole Bardi, Orazio Mandraffino, Oscar Maraldo, Pamela Raggi, Paolo Attolini, Pasquale Vaira, Pasquale Buonanno, Patricia Galán Menéndez, Patrizia Morettoni, Patrizio Papa, Paula Fernández-Valdes-Bango, Pier Paolo Gaglioti, Pilar cobeta, Placido Calì, Raffaele De Luca, Raffaele Russo, Giuseppe Rapisarda, Raquel Del Reino Iniesta, Raquel Estevez Martinez, Riccardo Colombo, Rita Perna, Romolo Villani, Rosalia Navarro Perez, Ruggero Vacirca, Salvatore Buscemi, Salvatore Napoli, Salvatore Sardo, Salvatrice Taravella, Sara Accetta, Sebastiana Saglimbene, Selenia Venere Lanza, Serena Ricalzone, Silvia Nardi, Simone Binda, Simone Grasso, Tiziana Costagliola, Tiziana Palladino, Tommaso Fossali, Valentina Arcidiacono, Valentina Bellini, Valentina Ceccarelli, Valentina Girotto, Valeria Camemolla, Valeria Drago, Valerio Manfrellotti, Vincenzo Francesco Tripodi, Vito Delmonte, Walter Gallese, Zaira Simonelli

**Affiliations:** 1 Division of Anesthesia and Intensive Care, Department of Human Pathology of the Adult and Evolutive Age “Gaetano Barresi”, Policlinico “G. Martino”, University of Messina, Messina, Italy; 2 Institute for Translational Medicine and Therapeutics, University of Pennsylvania Perelman School of Medicine, Philadelphia, PA 19104-5158 USA; 3 Outcomes Research Consortium, Cleveland, OH 44195 USA; 4 Dipartimento Area Emergenza Urgenza, Fondazione IRCCS Ca’ Granda - Ospedale Maggiore Policlinico, Milan, Italy; 5 Anesthesia and Intensive Care, San Martino Policlinico Hospital, IRCCS for Oncology and Neuroscience, Genoa, Italy; 6 Department of Surgical Sciences and Integrated Diagnostic (DISC), University of Genoa, Genoa, Italy; 7 Anesthesiology, Critical Care and Pain Medicine Division, Department of Medicine and Surgery, University of Parma, Parma, Italy; 8 Department of Anesthesia and Intensive Care Medicine, IRCCS Humanitas Research Hospital, Via Manzoni 56, Rozzano, Milan, 20089 Italy; 9 Department of Biomedical Sciences, Humanitas University, Via Rita Levi Moltancini 4, Pieve Emanuele, Milan, 20072 Italy; 10 Cardiothoracic and Vascular Anesthesia and Intensive Care, Department of Anesthesia and Critical Care Medicine, Azienda Ospedaliero Universitaria Pisana, Pisa, Italy; 11 Department Clinical Internal, Anesthesiological and Cardiovascular Sciences, University of Rome, “La Sapienza,” Policlinico Umberto Primo, Rome, Italy; 12 Consiglio Nazionale Delle Ricerche, CNR-IPCF, Messina, Italy; 13 Department of Anaesthesia and Intensive Care, “Policlinico-San Marco” University Hospital, Catania, Italy; 14 Department of Medicine, Surgery and Neuroscience, Anesthesia and Intensive Care Unit, University of Siena, Siena, Italy; 15 Unit of Anesthesia and Intensive Care, San Camillo-Forlanini Hospital, Rome, Italy; 16 Department of Surgical Oncological and Oral Science, University of Palermo, Palermo, Italy; 17 Department of Anesthesia Intensive Care and Emergency, Policlinico Paolo Giaccone, Palermo, Italy

**Keywords:** Blood pressure, Volume clamp, Postoperative complications, Intraoperative monitoring, Hemodynamic monitoring

## Abstract

**Background:**

Blood pressure has become one of the most important vital signs to monitor in the perioperative setting. Recently, the Italian Society of Anesthesia Analgesia Resuscitation and Intensive Care (SIAARTI) recommended, with low level of evidence, continuous monitoring of blood pressure during the intraoperative period. Continuous monitoring allows for early detection of hypotension, which may potentially lead to a timely treatment. Whether the ability to detect more hypotension events by continuous noninvasive blood pressure (C-NiBP) monitoring can improve patient outcomes is still unclear. Here, we report the rationale, study design, and statistical analysis plan of the niMON trial, which aims to evaluate the effect of intraoperative C-NiBP compared with intermittent (I-NiBP) monitoring on postoperative myocardial and renal injury.

**Methods:**

The niMon trial is an investigator-initiated, multicenter, international, open-label, parallel-group, randomized clinical trial. Eligible patients will be randomized in a 1:1 ratio to receive C-NiBP or I-NiBP as an intraoperative monitoring strategy. The proportion of patients who develop myocardial injury in the first postoperative week is the primary outcome; the secondary outcomes are the proportions of patients who develop postoperative AKI, in-hospital mortality rate, and 30 and 90 postoperative days events. A sample size of 1265 patients will provide a power of 80% to detect a 4% absolute reduction in the rate of the primary outcome.

**Conclusions:**

The niMON data will provide evidence to guide the choice of the most appropriate intraoperative blood pressure monitoring strategy.

**Clinical trial registration:**

Clinical Trial Registration: NCT05496322, registered on the 5^th^ of August 2023.

## Background

In the last 50 years, anesthesia-related mortality has decreased dramatically [1], with a European in-hospital mortality rate after noncardiac surgery of 4% [2]. The relationship between intraoperative arterial blood pressure targets and serious perioperative complications is of critical importance in perioperative medicine. Hypertension and hypotension can affect the function of vital organs such as the brain, heart, and kidneys, and recent studies have demonstrated associations between low mean arterial pressure (MAP), organ injury, and 30-day mortality [3–9]; however, this finding has been questioned by a recent meta-analysis [10]. Blood pressure (BP) has become one of the most important vital signs to be monitored in the perioperative setting. Recently, the Italian Society of Anesthesia Analgesia Resuscitation and Intensive Care (SIAARTI) recommended, with a low grade of evidence, continuous BP monitoring during the intraoperative period [11]. Although BP assessment is mandatory and part of the standard monitoring in the perioperative setting, there is currently no consensus on specific BP targets for different surgical patient populations. Furthermore, the depth of hypotensive events per se and the total time spent in hypotension can be associated with worse outcomes [8, 12].

In recent years, there has been a trend toward keeping MAP higher than 65 mmHg to reduce postoperative complications [8]. A personalized intraoperative BP management strategy, considering the individual patient’s preoperative BP, showed an improvement in postoperative outcomes [13]. This strategy is still debated compared to those based on absolute intraoperative MAP thresholds [9].

Intraoperative blood pressure is usually measured intermittently using non-invasive oscillometric devices (I-NiBP) every 3–5 min, potentially leading to unrecognizable periods of hypotension [14]. Less than 5 min of MAP less than 55 mmHg during noncardiac surgery is independently associated with an increased risk (30%) of postoperative acute kidney injury (AKI) and myocardial injury [8]. On the contrary, continuous monitoring allows early detection of hypotensive events and probably a prompter treatment. Commonly, invasive blood pressure monitoring, which provides continuous blood pressure, is used in selected patients at high risk of complications and/or major surgeries. In patients at lower risk, continuous non-invasive blood pressure (C-NiBP) monitoring with the finger-cuff method may be of potential benefit. These devices adopt the volume-clamp [15, 16] method and the physiocal algorithm for continuous BP estimation (ClearSight® Edwards Lifesciences Corp, Irvine, CA), using a cuff wrapped around the middle phalange of the finger. Evidence suggests that arterial pressure measurements from this monitor are comparable to invasive blood pressure monitoring [17–19] and non-inferior to conventional automatic oscillometric methods [20].

The use of C-NiBP monitoring is associated with a shorter duration of intraoperative hypotension and hypertension compared to conventional intermittent blood pressure monitoring [21–23]. Chen et al. demonstrated that using C-NiBP devices for every hour of surgery, an average of 14 min of potentially treatable hypotensive events [13]. Whether the ability to detect more hypotension events by C-NiBP monitoring can improve patients’ outcomes is still unclear. Therefore, we designed a randomized trial to evaluate the effect of intraoperative C-NiBP compared with I-NiBP monitoring on postoperative myocardial injury.

## Main text

### Objectives

We hypothesize that C-NiBP monitoring could reduce postoperative complications, decreasing the duration and number of intraoperative hypotension events. The primary aim of the Continuous vs intermittent Non-Invasive blood pressure MONitoring (NiMon) trial is to evaluate whether the use of C-NiBP as compared to I-NiBP monitoring during the intraoperative period decreases the proportions of patients who develop myocardial injury in the first postoperative week.

### Trial design

The niMon trial is an investigator-initiated, multicenter, international, open-label, parallel-group, randomized clinical trial.

## Methods

### Study setting

Potential participants will be screened in Europe and recruited in participating centers by the local investigators. Each participating center will be coordinated by a local principal investigator who is responsible for trial activities, local monitoring, and adherence to regulations at each site. The complete recruiting centers list is reported on the trial website (http://www.nimontrial.it).

### Eligibility criteria

#### Inclusion criteria

- All adult (age ≥ 18 years) patients scheduled for elective noncardiac;
- Surgical cases planned with non-invasive BP monitoring according to local clinical practice or policies.

#### Exclusion criteria

- Missing preoperative serum creatinine during the 30 days prior to surgery;
- Preoperative dialysis;
- Chronic kidney disease (eGFR < 60 ml/min Cockcroft-Gault equation);
- Surgical procedure with expected duration lasting less than 60 min;
- Planned use of permissive hypotension;
- Nephro/urological procedures (including nephrectomy and renal transplantation, pheochromocytoma surgery);
- Raynaud’s syndrome; and
- Refusal to give informed consent.

### Intervention description

During the 30 days before a scheduled surgery, all patients will be screened for inclusion criteria, and reassessed the day of surgery; after obtaining written informed consent, the patients will be randomized to a blood pressure measurement strategy (C-NiBP or I-NiBP), once arrived in the operating room. Thereafter, patient monitoring is completed according to local policy. Patients may receive general or regional anesthesia according to the preferences of the attending anesthesiologist, using the drugs commonly used in local practice.

Mechanical ventilation, when necessary, is delivered with the use of a tidal volume of 6–8 ml/kg predicted body weight and a PEEP level between 5 and 8 cmH_2_O. The FiO_2_ is adjusted to obtain an oxygen saturation > 95%. The respiratory rate is set to maintain end-tidal carbon dioxide between 30 and 35 mmHg. The core temperature should be maintained at least 36 °C. In each group, patients receive balanced crystalloid solutions at 3–5 ml/kg per hour as maintenance fluid during surgery. In both groups, the fluid balance is titrated by the attending physician, according to local good practice. In both groups, every hypotensive event (*defined as MAP* < *65 mmHg for at least 1 min*) should be promptly treated, with the most appropriate treatment as decided by the attending anesthetist. At any time, the treating physician could choose to start invasive arterial BP monitoring.

### Outcomes

The study has one primary and several secondary outcomes that are described below.

#### Primary outcome

1. Proportions of patients developing myocardial injury after noncardiac surgery (MINS) in the first postoperative week.
  *Definitions*: elevation of serum troponin T (TnT) >= 30 ng/L or high-sensitivity troponin T (hsTnT) >20 ng/L to <65 ng/L with an absolute change of ≥5 ng/L from baseline, or a high-sensitivity troponin T concentration ≥65 ng/L in the first postoperative week

#### Secondary outcomes

1. Proportions of patients who develop postoperative AKI in the first postoperative week.
  *Definition:* increase in postoperative serum creatinine concentration during the first 7 postoperative days by more than 1.5-fold or greater than 0.3 mg/dl or urine volume < 0.5 mL/kg/h for 6 h. Baseline creatinine concentration was defined as the most recent recorded measurement within 30 days before the surgery.
2. In-Hospital Mortality
  *Definition: Death for any-cause during postoperative hospital stay*
3. Proportion of severe clinical events occurring within the 30 days following surgery
  *Definition: Stroke, Nonfatal cardiac arrest, AKI Stage 2–3, Sepsis, Death*
4. Proportion of severe clinical events occurring within the 90 days following surgery
  *Definition: Stroke, Nonfatal cardiac arrest, AKI Stage 2–3, Sepsis, Death*

### Assignment of interventions

Eligible patients will be randomized in a 1:1 ratio to receive C-NiBP or I-NiBP as an intraoperative monitoring strategy. A computer-generated permuted block randomization list with varying block sizes will be concealed and created by a statistician. Randomization will be implemented using a web-based electronic system incorporated in the electronic case report form to ensure allocation concealment. Randomization will be stratified according to the patient cardiovascular perioperative risk (RCRI). Trial intervention is not blinded for investigators in the operating room and patients because blinding to intraoperative blood pressure monitoring is unfeasible. The statistician will be blinded to allocation. Clinical outcomes will be recorded by personnel blinded to patient allocation according to planned timepoints (preoperative, intraoperative, daily up to 7 days following surgery, hospital discharge, 30 and 90 days following surgery).

## Data collection and management

This study will use Research Electronic Data Capture (REDCap), owned by the SIAARTI, for data collection, transmission, and storage. All study data will be entered via a 2-factor password-protected website. REDCap servers are hosted in a data center inside the European Union, and all web-based information transmission is encrypted. The study data manager will assess the quality of the data (validation of range and consistency). At the end of each center's enrollment period, specific queries will be sent to each center by the study's data manager, along with the members of the Steering Committee. After recording all entries and clarifying all queries, the database will be closed before the data analysis.

### Sample size

Previous data indicate that the incidence of myocardial injury after noncardiac surgery (MINS), according to the definition of TnT elevation > 30 ng/L, is 7.9% [24]. We calculated that the enrollment of 602 patients per group (total: 1204 patients) would provide 80% power to detect a 4% absolute reduction (50% relative risk reduction, number needed to treat of 25) in the primary outcome rate in either the C-NIBP or I-NIBP group, with an alpha level of 0.05. In the occurrence that most centers will have employed the high sensitivity troponin assay (MINS incidence 17%) [25], we plan to evaluate the appropriateness of the sample size during the interim analyses, considering the incidence of the primary outcome.

The attrition rate is expected to be less than 5% and likely due to protocol violations, crossover, and drop-outs. We plan to enroll a total of 1265 patients in 24 months.

### Statistical method, analysis strategy, subgroup analysis

The Kolmogorov–Smirnov test and histogram visualization will be used to evaluate the distribution of the variables and descriptive statistics, including mean (± standard deviation) or median and interquartile range, will be presented based on the skewness of the distribution. Categorical variables will be presented as proportions. Comparison between groups will be conducted using Student’s *t* test for normally distributed continuous variables, the Mann–Whitney-Wilcoxon nonparametric test for non-normally distributed variables, and the chi-square test or Fisher’s exact test for discrete variables. A preplanned subgroup analysis will be performed on the surgical risk stratification (RCRI), fluid balance strategy, and hypertension history. All analyses will be performed on an intention-to-treat (ITT) basis. All statistical tests will be two-sided and assume a significance α-level of 0.05.

### Safety monitoring

An independent Trial Oversight Committee has been established, consisting of independent clinicians who have experience in clinical monitoring devices and in the conduct, monitoring, and analysis of data from randomized trials. A single interim analysis will be performed when a number of patients equal to two-thirds of the predefined sample (843/1265) are randomized and completed the outcome up to hospital discharge. We will consider as early stopping criteria a predetermined 2-sided *p* value < 0.01 for the rejection of the null hypothesis that the two BP monitoring strategies are equivalent in terms of primary outcome rate.

## Ethics and dissemination

### Ethics and protocol registration

The trial protocol was prepared according to the SPIRIT (Standard Protocol Items: Recommendations for Interventional Trials) recommendations [26], following the European Clinical Trial Directive (Directive 2001/20/EC) and the Good Clinical Practice Directive (Directive 2005/28/EC), and Italian current regulations.

The Ethical Committee of the coordinator center (Comitato etico interaziendale—Messina) approved the protocol study (Protocol Number 16/22 2—Approval date 19/7/22).

The trial protocol has been prospectively registered (Clinical Trial Registration: NCT05496322) by the principal investigator (AN).

Each investigator will have to assess whether the study protocol requires formal approval by its Ethics Committee of Reference. This is a low/minimal-risk study. Only deidentified data will be recorded, and patient data in the database will not be able to be linked to the patients. No data present in the medical/outpatient records will be removed, altered, or modified. Regarding the protection of privacy, it must be considered that physicians are bound by professional secrecy.

### Confidentiality

Subject confidentiality is strictly held in trust by the participating investigators, research staff, and sponsoring institution. The study protocol, documentation, data, and all other information generated will be held in strict confidence. All the data reported in the eCRF are anonymized (number of center-number patients). No information concerning this study or the data will be released to any unauthorized third party without prior written approval of the study Management Committee.

All local legal requirements regarding data protection will adhere to EU-GDPR. All study findings and documents will be regarded as confidential. The investigator and research team members must not disclose any information without prior written approval from the sponsor.

The pseudonymity of participating patients must be maintained. The patients will be recognized on CRFs and other documents by an identification number throughout documentation and evaluation. Records that identify the patient personally (e.g., the signed informed consent) must be maintained in confidence by the local investigators. The patients will be told that all study findings will be stored on a computer and handled with the strictest confidence.

### Sponsor/founder

This study was partly supported by a grant for scientific research from the SIAARTI. Where needed, SIAARTI will provide recruiting centers, data transfer agreements, and help in submission of the study documentation to the research ethics committees. The study is investigator-initiated and not funded by any industry-related external body. There will be no monetary incentives or reimbursement for participants and investigators of participating centers. No funding or external support will be provided to the study centers for the devices involved in the trial. The funder is not involved in study design, data collection and interpretation, manuscript writing, and in the decision to submit the manuscript for publication.

### Dissemination plan

The Steering Committee will conduct the synthesis and statistical analysis of the data and produce the manuscript draft. Group authorship (“SIAARTI Study Group”) will be created, which will include all the investigators of the participating centers. The final publication of the data, in the form of an article published in a peer-reviewed journal, will take into account the participation of all members of the SIAARTI Study Group whose names will be traceable through scientific databases (PubMed, Scopus).

### Provisions for post‐trial care

Patient participation is covered by insurance as per legal requirements in Italy. The insurance applies to the damage that becomes apparent during the study only for the Italian recruiting centers.

### Adverse event reporting and harms

Although serious adverse events are not to be expected, the sponsor and the principal investigator will suspend the study if there is reasonable doubt that the continuation of the study will compromise the safety and well-being of participants. In this case, the local ethics committee will be informed immediately, and the study will remain suspended pending a favorable decision from the local ethics committee.

Adverse events will be noted, and serious adverse events will also be reported to the local ethics committee.

## Discussion

The niMON trial is an investigator-initiated, randomized multicenter trial with a pragmatic design. The non-invasive BP monitoring strategy during anesthesia may play a role in the outcome of low-to-mid-risk patients. The C-NiBP monitoring reduces hypotension events compared to I-NiBP monitoring during induction of anesthesia [22] and intraoperative period [21, 22]. Whether the reduction of hypotension events decreases the rate of perfusion-related postoperative complications remains elusive. Therefore, we designed the niMON trial to test the hypothesis that finger-cuff-based C-NiBP monitoring has the potential to improve perioperative outcomes.

The occurrence of myocardial injury after noncardiac surgery and acute kidney injury are the most clinically relevant perfusion-related complications associated with intraoperative hypotension events. The heterogeneous definition of perioperative myocardial injury [8, 9] is an open topic; nevertheless, it is independently associated with adverse outcomes. The data gathered with the niMON trial will also be useful to support and improve the practice of perioperative TnT measurements.

The niMON data will provide evidence to guide the choice of the most appropriate intraoperative blood pressure monitoring strategy.

## Data Availability

Not applicable.

## Abbreviations

MAP: Mean arterial pressure
BP: Blood pressure
C-NiBP: Continuous non-invasive blood pressure
I-NiBP: Intermittently non-invasive blood pressure
AKI: Acute kidney injury
PEEP: Positive end-expiratory pressure
MINS: Myocardial injury after noncardiac surgery
TnT: Serum troponin T
hsTnT: High-sensitivity troponin T
RCRI: Revised cardiac risk index
