# Correction: Continuous vs intermittent Non-Invasive blood pressure MONitoring in preventing postoperative organ failure (niMON): study protocol for an open-label, multicenter randomized trial

**DOI:** 10.1186/s44158-024-00151-9

**Published:** 2024-02-22

**Authors:** Alberto Noto, Athanasios Chalkias, Fabiana Madotto, Lorenzo Ball, Elena Giovanna Bignami, Maurizio Cecconi, Fabio Guarracino, Antonio Messina, Andrea Morelli, Pietro Princi, Filippo Sanfilippo, Sabino Scolletta, Luigi Tritapepe, Andrea Cortegiani, SIAARTI Study Group

**Affiliations:** 1 Division of Anesthesia and Intensive Care, Department of Human Pathology of the Adult and Evolutive Age “Gaetano Barresi”, Policlinico “G. Martino”, University of Messina, Messina, Italy; 2 Institute for Translational Medicine and Therapeutics, University of Pennsylvania Perelman School of Medicine, Philadelphia, PA 19104-5158 USA; 3 Outcomes Research Consortium, Cleveland, OH 44195 USA; 4 Dipartimento Area Emergenza Urgenza, Fondazione IRCCS Ca’ Granda - Ospedale Maggiore Policlinico, Milan, Italy; 5 Anesthesia and Intensive Care, San Martino Policlinico Hospital, IRCCS for Oncology and Neuroscience, Genoa, Italy; 6 Department of Surgical Sciences and Integrated Diagnostic (DISC), University of Genoa, Genoa, Italy; 7 Anesthesiology, Critical Care and Pain Medicine Division, Department of Medicine and Surgery, University of Parma, Parma, Italy; 8 Department of Anesthesia and Intensive Care Medicine, IRCCS Humanitas Research Hospital, Via Manzoni 56, 20089 Rozzano, Milan Italy; 9 Department of Biomedical Sciences, Humanitas University, Via Rita Levi Moltancini 4, Pieve Emanuele, 20072 Milan, Italy; 10 Cardiothoracic and Vascular Anesthesia and Intensive Care, Department of Anesthesia and Critical Care Medicine, Azienda Ospedaliero Universitaria Pisana, Pisa, Italy; 11 Department Clinical Internal, Anesthesiological and Cardiovascular Sciences, University of Rome, “La Sapienza,” Policlinico Umberto Primo, Rome, Italy; 12 Consiglio Nazionale Delle Ricerche, CNR-IPCF, Messina, Italy; 13 Department of Anaesthesia and Intensive Care, Policlinico-San Marco” University Hospital, Catania, Italy; 14 Department of Medicine, Surgery and Neuroscience, Anesthesia and Intensive Care Unit, University of Siena, Siena, Italy; 15 Unit of Anesthesia and Intensive Care, San Camillo-Forlanini Hospital, Rome, Italy; 16 Department of Surgical Oncological and Oral Science, University of Palermo, Palermo, Italy; 17 Department of Anesthesia Intensive Care and Emergency, Policlinico Paolo Giaccone, Palermo, Italy

**Correction: J Anesth Analgesia Crit Care 4, 7 (2024)**

**https://doi.org/10.1186/s44158-024-00142-w**

The original publication of this article contained an incorrect author name in the SIAARTI Study Group. The incorrect and correct information is listed in this correction article. The original article has been updated.

Incorrect

Vincenzo Tripodo

Correct

Vincenzo Francesco Tripodi

## References

[1] Noto A (2024). Continuous vs intermittent Non-Invasive blood pressure MONitoring in preventing postoperative organ failure (niMON): study protocol for an open-label, multicenter randomized trial. J Anesth Analgesia Crit Care.

